# Improving Transferability of Introduced Species’ Distribution Models: New Tools to Forecast the Spread of a Highly Invasive Seaweed

**DOI:** 10.1371/journal.pone.0068337

**Published:** 2013-06-28

**Authors:** Heroen Verbruggen, Lennert Tyberghein, Gareth S. Belton, Frederic Mineur, Alexander Jueterbock, Galice Hoarau, C. Frederico D. Gurgel, Olivier De Clerck

**Affiliations:** 1 School of Botany, University of Melbourne, Parkville, Victoria, Australia; 2 Phycology Research Group, Ghent University, Ghent, Belgium; 3 Flanders Marine Institute (VLIZ), Ostend, Belgium; 4 School of Earth and Environmental Sciences, University of Adelaide, Adelaide, South Australia, Australia; 5 Mediterranean Institute of Oceanography (MIO), Aix-Marseille University, Marseille, France; 6 Marine Ecology Group, University of Nordland, Bodø, Norway; 7 South Australia State Herbarium, Department of Environment Water and Natural Resources, Adelaide, South Australia, Australia; 8 South Australia Research and Development Institute (Aquatic Sciences), Henley Beach, South Australia, Australia; Manchester University, United Kingdom

## Abstract

The utility of species distribution models for applications in invasion and global change biology is critically dependent on their transferability between regions or points in time, respectively. We introduce two methods that aim to improve the transferability of presence-only models: density-based occurrence thinning and performance-based predictor selection. We evaluate the effect of these methods along with the impact of the choice of model complexity and geographic background on the transferability of a species distribution model between geographic regions. Our multifactorial experiment focuses on the notorious invasive seaweed *Caulerpacylindracea* (previously *Caulerpa* *racemosa* var. *cylindracea* ) and uses Maxent, a commonly used presence-only modeling technique. We show that model transferability is markedly improved by appropriate predictor selection, with occurrence thinning, model complexity and background choice having relatively minor effects. The data shows that, if available, occurrence records from the native and invaded regions should be combined as this leads to models with high predictive power while reducing the sensitivity to choices made in the modeling process. The inferred distribution model of *Caulerpacylindracea* shows the potential for this species to further spread along the coasts of Western Europe, western Africa and the south coast of Australia.

## Introduction

Species distribution models (SDMs) help us understand and map species’ distributions, play a key role in forecasting range expansion of introduced species and can help us predict the effects of climate change on species distributions [1-4]. An SDM characterizes the species’ response to relevant environmental variables, using either physiological information from experimental work (mechanistic models) or by relating the presence and/or absence of the species to environmental information (correlative models) [5]. This response is subsequently projected into geographic space using gridded environmental layers, resulting in a map showing the potential distribution of the species. Because experimental physiological work has not been carried out for a great majority of species, correlative approaches dominate species distribution modeling. Furthermore, it is quite troublesome to assess the absence of species from an area while species occurrence data are abundant in museum databases and the literature. As a consequence, most SDMs rely on presence-only techniques [1].

A crucial assumption in using SDMs to forecast the spread of introduced species or distribution changes in response to environmental change is that the model is transferable to the new conditions [6]. In the case of introduced species, models trained primarily on distribution data from the species’ native range need to be transferred to the region where it has been introduced. This often implies projecting the species response to climatic conditions that are not present in the native (training) range, which is an innately difficult task. For such situations, it is valuable to visualize those areas where extrapolation beyond observed conditions was required and consider those distribution predictions as uncertain [7,8]. In addition, the ability of presence-only methods to capture a species’ ecological response is affected by the choice of background points [7], predictor variables [9], model complexity [10,11] and the geographic spread of occurrence records in relation to environmental gradients [12,13]. Besides these problems, it is also possible that biotic interactions limit the utility of models based on abiotic predictors [14] and, of course, there is always the possibility that the fundamental niche of the introduced population has changed due to natural selection [15,16].

This study focuses on the choices made during the modeling process that affect the transferability and overall predictive performance of the resulting model. We introduce two new methods that have the potential to increase the transferability of correlative SDMs: density-based occurrence thinning and performance-based predictor selection. As a case study, we apply these to the highly invasive seaweed species, *Caulerpacylindracea* , in order to assist in assessing the risk of further spreading as well as predicting areas with suitable environmental conditions worldwide.

## Methods

### Experimental Design

The overarching goal of the present study is to examine and improve the overall performance and the transferability between regions of maximum entropy (Maxent) presence-only models of introduced species. The experimental design centers on the impact of four important choices that have to be made during the modeling process: (1) the amount of geographic autocorrelation in occurrence records, (2) the choice of predictor variables, (3) the complexity of the model, and (4) the selection of background points.

Because most environmental variables show spatial autocorrelation, geographically biased sampling of occurrence records (e.g. heterogeneous accessibility and local expertise) naturally results in environmental biases in the data used to train the SDM, leading to model misspecification [12,17] and issues related to its evaluation [18]. We introduce a method that thins occurrence records in densely sampled regions to obtain a more even geographic distribution (details given below). To examine the effect of this method, models with and without occurrence thinning are compared.

The choice of predictor variables is arguably one of the most studied elements affecting the transferability of SDMs, with several papers showing differences in transferability depending on which predictor set is used [9,19,20]. This has also led to the recognition of predictor variables as more conserved or relaxed, depending on whether they match between native and invaded species occurrences or not [9,21]. We introduce a method that surveys the performance of all possible predictor sets (explained below) and evaluate the transferability between regions of models built with two different sets of predictors.

The complexity of an SDM is also known to impact on its predictive performance, with overfitting often leading to poor transferability [10,22,23]. By default, Maxent determines the types of features it allows automatically, based on the number of samples available for model training [24], but this standard behavior has been reported to result in overfitted models [11]. We compare models with automatically determined model complexity to models forced to be simple.

Finally, the selection of background points is known to affect the outcome of presence-only SDMs [7,25,26]. To examine this, we compare SDMs built with global background points to models built with a regional background.

Using *C* *. cylindracea* as a case study, model transferability was assessed by training models on samples from either the native or the invaded range and measuring the overlap of the two models, as well as by calculating how well they predict presences in the other range. We also compare the overall predictive performance of SDMs trained with occurrences from either range to that of models combining occurrences from both ranges.

### Study Species and Environmental Data

This study focuses on the introduced and highly invasive seaweed species *Caulerpacylindracea* Sonder [27]. Specimens of the *Caulerpa* genus are well known for their rampant morphological plasticity that, due to the inconsistent use of varieties and forms amongst taxonomists, has resulted in a confusing nomenclature. Most of this confusion has existed around the *C* *. racemosa* /*peltata* complex that has more than 30 described varieties and forms [28]. Until recently this included *C* *. cylindracea* , which, although originally described as an independent species, had long been considered a form of *C* *. racemosa* var. *laetevirens* until it was raised to varietal status [29] and it is now due to be reinstated as an independent species [28].

Since the early 1990s *C* *. cylindracea* has rapidly and aggressively spread in the Mediterranean Sea and Canary Islands, representing one of the most dramatic marine invasions in terms of establishment and ecological dominance [30,31]. The species has been reported from all kinds of substrata and depths, as part of a variety of benthic assemblages, and thrives in disturbed habitats of the heavily urbanized Mediterranean coastlines [30,32]. Invasive populations of *C* *. cylindracea* establish dense and compact monospecific stands, which easily overgrow and outcompete and/or negatively impact other seaweed [33,34], seagrass [35] and invertebrate species [36,37] leading to biotic homogenization [38] and an overall decrease of species diversity in affected areas [30]. To date only partial recovery of the assemblages could be observed after eradication of *C* *. cylindracea* in Italy and France [33,39].

Unlike *C* *. taxifolia* , which was accidentally introduced from a public aquarium [40], the vector of introduction of *C* *. cylindracea* to the Mediterranean Sea is unknown. It was initially hypothesized to be a Lessepssian immigrant [41,42], or a hybrid between *C* *. racemosa* var. *turbinata* and an unknown tropical variety [43], until molecular investigations identified a potential source population in southwestern Australia [29]. However, recent findings indicate that the native range of *C* *. cylindracea* is much larger than previously thought (extending from Western Australia around northern Australia into the Great Barrier Reef and New Caledonia), and that the source of the invasive *C* *. cylindracea* populations in the Mediterranean Sea is not known with certainty [44].

Whatever the vector and source population, *C* *. cylindracea* is spreading rapidly with reports of its presence in 12 Mediterranean countries including all the large islands [29,45], and has more recently been reported from two locations on the southern coast of Australia (Adelaide, SA and Portland, VIC, e.g. references 46,47 and unpublished data GSB). As *C* *. cylindracea* is only found near shipping ports and had not been reported from this area prior to 2003 [48], it is most likely that this species is a recent introduction. The rapid spread of this species through the European invaded range makes it a suitable case study for the question at hand.

A total of 191 distribution records were assembled from the native range in and around Australia (65 records), the invaded range in Europe (111) and the recently invaded areas in southern Australia (15). The data sources for these records are: Australia Virtual Herbarium (http://chah.gov.au/avh/), new collections from Victoria by GSB deposited in the AD herbarium, the data gathered by FM for the ERC FP5 ALIENS project, and the literature [31,41,44,48-60]. The absence of the species in various DNA bar coding surveys of *Caulerpa* from some other parts of the Indo-Pacific (Philippines, Japan, Tanzania, Red Sea) suggests that the native range may be limited to Australia and some closeby locations (unpublished data: Stefano Draisma, Thomas Sauvage, Heroen Verbruggen).

We used the Bio-ORACLE dataset [61] as a source of marine environmental grids (90^°^ N–90ºS, real values). To make the distribution records compatible with the grids, occurrence coordinates situated on land according to the Bio-ORACLE grids were moved to the closest cell in the ocean. When multiple records were situated in the same Bio-ORACLE grid cell, a single record was retained and as a result, the dataset reduced to 95 distribution records.

### Occurrence Thinning

Geographical biases in the occurrence records were dampened by thinning the distribution points with OccurrenceThinner 1.03 [62]. We developed this program to filter occurrence records using a probability-based procedure. The probability that any specific occurrence record is removed is proportional to the density of occurrence records in the area as defined by a kernel density grid. The two-dimensional binned kernel density grid used in this procedure was computed from the occurrence records with the bkde2D function in the R package KernSmooth v.2.23-7 [63,64], with a bandwidth of 3.0. The thinning procedure with thresholds t_1_=0.5 and t_2_=1.0 was repeated 10 times, resulting in 10 occurrence-thinned datasets. These datasets had on average 25 records from the native range, 46 from the European invaded range, and three from the southern Australian invasive populations.

To evaluate whether occurrence thinning influences model transferability and performance, we compared Maxent models based on a thinned subset of samples with models using all occurrence records (but limited to one per cell as mentioned above).

### Predictor Sets

The predictor variables were chosen in two steps. The first step consisted of *a priori* selection of a set of 8 predictors. This selection was based on knowledge of the physiological determinants of seaweed distributions [65], and takes the structure of the Bio-ORACLE dataset into account by not using multiple closely correlated predictors. The eight resulting predictors were mean sea surface temperature (SSTmean), the range in sea surface temperature (SSTrange) as a measure of seasonality, mean photosynthetically active radiation (PARmean), salinity, pH, mean diffuse attenuation (DAmean) as a measure of water transparency, dissolved oxygen (dissox) and the phosphate concentration. Nitrate concentration was not included because it is correlated with the phosphate concentration [61].

In the second step, the predictive ability of those eight variables was explored using Maxent Model Surveyor (MMS) version 1.03 [66]. We developed this software to evaluate the performance of all possible subsets of variables (2^8^ - 1 = 255 for our eight predictors), using the test AUC (Area Under the receiver operating characteristic Curve) to measure model performance [67]. The program was run multiple times: (1) on samples from native range with global background, (2) on samples from invaded range with global background, (3) on samples from both ranges with global background, (4) on samples from native range with background restricted to native range, and (5) on samples from invaded range with background restricted to invaded range. The program used 50% of the samples for training and 50% for testing. It worked from the thinned set of occurrences and restricted the model complexity to linear and quadratic features. Each of the five runs listed above was repeated ten times (i.e., on each of the ten replicate sets of thinned occurrences). The training and test data were randomly drawn from the occurrence records and do not represent a subdivision into the native vs. invaded ranges. As a consequence, the model performance used to evaluate predictor combinations does not represent transferability between regions. From the MMS results, a consensus was derived as to which variables are most important across the different runs. We retained only those variables that were present in more than 60% of the top-scoring models for at least two out of three regions (native, Europe, combined, i.e. conditions 1, 2 and 3 described above). The 60% threshold criterion is essentially arbitrary – we chose it because it halved the number of predictor variabes from eight to four (specified in results). Retaining variables important in at least two regions was done because it would prefer variables of global, rather than regional, relevance.

In order to evaluate whether this predictor selection approach can improve the transferability of models across regions, Maxent models were run with all eight variables listed above as well as the subset of four variables generated with the predictor selection procedure.

### Model Complexity

Model complexity was varied to verify its impact on the predictive power and transferability. The first condition used the default behavior of Maxent (auto-features), which determines which features are used based on the number of samples [24]. The second condition forced the use of smooth response curves by allowing only linear and quadratic features to be fitted.

### Background Selection

Data for background points was extracted from the Bio-ORACLE grids [61]. Three sets of 10,000 random background points were created: (1) from the entire globe, (2) from the native range defined as a box around Australia with latitude between 5^°^ S and 45^°^ S and longitude between 100^°^ E and 175^°^ E, and (3) from the invaded range defined as western Europe extending to Africa and the Mediterranean Sea, between latitude 20^°^ N and 60^°^ N and longitude 35^°^ W and 40^°^ E. These boxes roughly correspond to the maps of the native and invaded ranges presented in the results. In each of these three cases, the background selection corrected for unequal areas at different latitudes (i.e. they correspond to random pixel draws from equal area grids).

To compare the effect of background selection on transferability, regional models with corresponding regional backgrounds were compared to regional models with global background. Models trained with combined samples from the native and invaded ranges always used the global background.

### Niche Model Inference

Niche models were inferred with Maxent 3.3.3f [24,68,69]. The analyses were automated via a Perl script and carried out on a multicore linux server. All analyses were run with 10,000 random background points as specified above. The training, test and background points, were provided as SWD files and the resulting models were projected onto the Bio-ORACLE grid [61]. Maxent’s jackknife function was activated and samples were not added to the background to avoid complicating model comparisons. The models resulting from the ten replicate occurrence-thinned training sets were averaged for visualization but other interpretations were based on the individual models.

### Downstream Analyses

Models were compared to identify which choices lead to better-performing models. In order to evaluate the transferability of models, we compared models built on the native and invaded ranges in a pairwise fashion, using the Schoener’s D niche similarity measure [70] and reciprocal test AUC (i.e. native training samples with test samples in invaded range and vice versa). The overall predictive power of models was compared with the test AUC, taking care to only compare models built with identical geographic background datasets.

## Results

### Exploration of new methods

**Figure 1:**
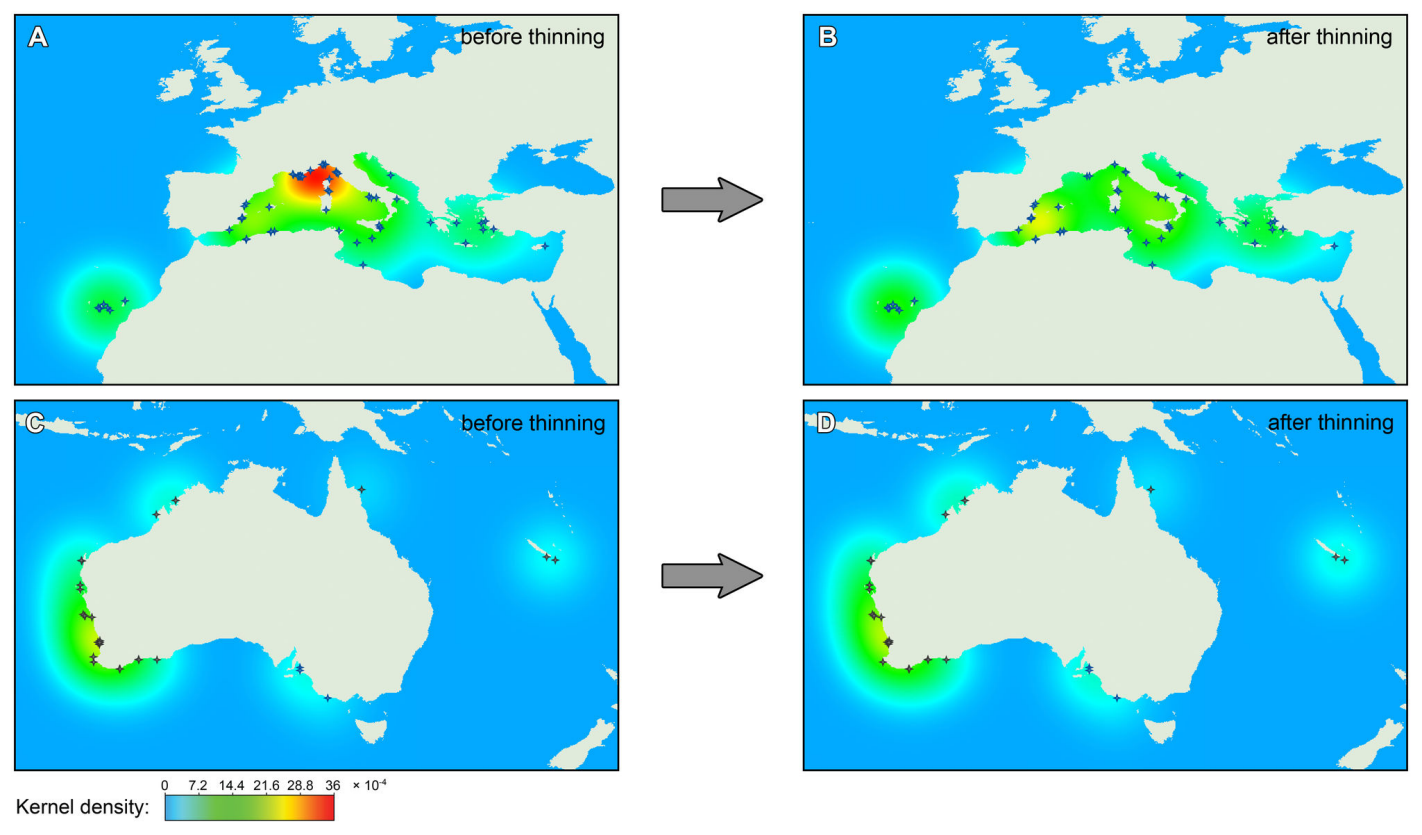
Effect of occurrence thinning on geographical sample bias. The colors on the map represent the regional sampling density, warmer colors indicating higher sample densities. Occurrence thinning substantially reduces the geographic sampling bias, as illustrated by the disappearance of the red blob along the French Riviera and closeby localities (panel A → B). There is less geographic sampling bias in the native range, so occurrence thinning does not have a big influence on the kernel density maps of that region (panel C → D). Note that the slightly elevated density close to the Spanish-French border in the Bay of Biscay (panels A and B) is caused by samples in the Mediterranean of which the kernel extends across land; there are no occurrences of *C* *. cylindracea* known from that area.

We implemented two methods that tackle issues related to the overall quality and transferability of niche models. The first of these, occurrence thinning, clearly reduced the geographic sampling bias present in the occurrence points, as indicated by the kernel density plots before and after occurrence thinning (Figure 1). In this figure, the red blob with dense sampling along the French Riviera and nearby localities disappears entirely after the thinning procedure (Figure 1A-B). Geographic sampling bias was less of a problem in the native range (Figure 1C-D).

**Figure 2:**
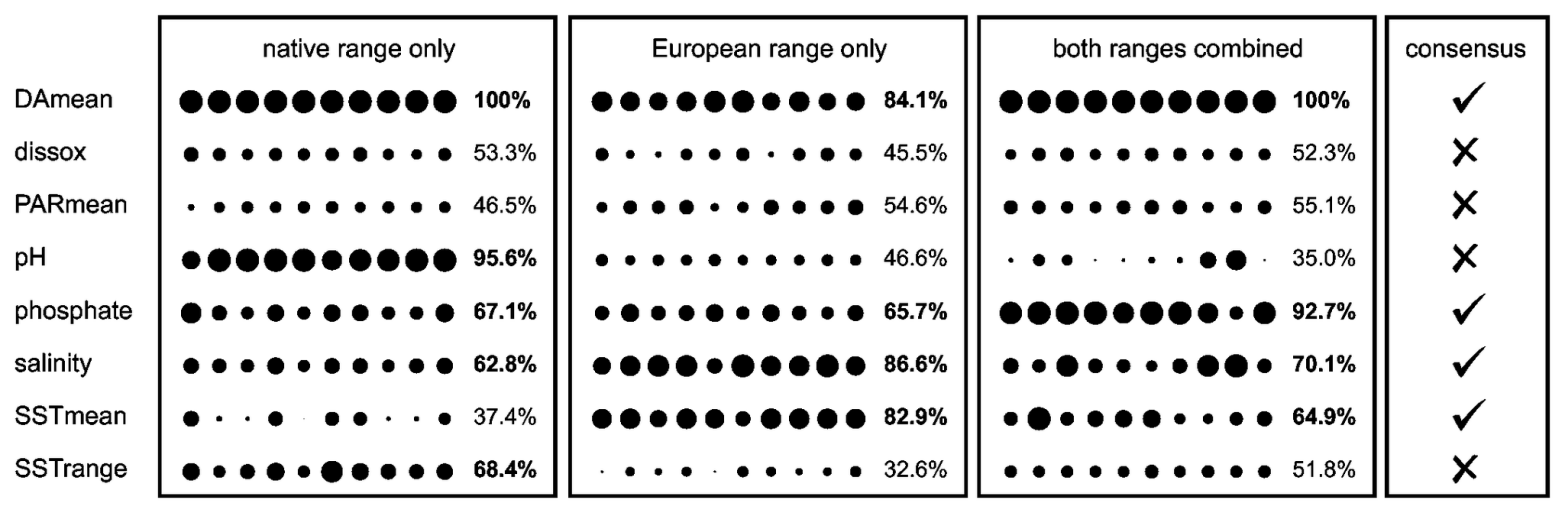
Results of the surveying procedure to identify the predictors present in top-scoring models. Each box contains the results of the survey for occurrence records from the native range, the invaded European range, or both ranges combined. Each column within a box represents a single survey carried out on one set of thinned coordinates. The circle diameter represents how often the variable in question occurred in the top 10 highest-scoring models (test AUC) for that set of thinned occurrences. The representation of each predictor in the top 10 is also summarized across columns (percentage indicates how many of the top 10 models had the predictor), and the consensus predictor set across ranges is indicated in the box on the right.

The results of the second method, which surveyed all combinations of predictor combinations, is summarized in Figure 2. As could be anticipated from previous studies, the representation frequency of variables among the top-scoring models is sensitive to whether the analysis was done on the native range, the invaded range, or both combined. Using local or global background points resulted in qualitatively similar results (Figure S1). The consensus made across the three boxes in Figure 2, including only variables that are likely to be of global significance (present in at least 60% of the top-scoring models for at least 2 out of 3 regions), consisted of 4 predictors: DAmean, phosphate, salinity and SSTmean.

The effect of these two methods on model performance was evaluated by including them as factors in our experimental design. So all Maxent analyses were run with all samples and thinned samples. Similarly, models were run with all eight variables included and with only the four consensus variables selected from the survey.

### Transferability as a function of modeling choices

**Figure 3:**
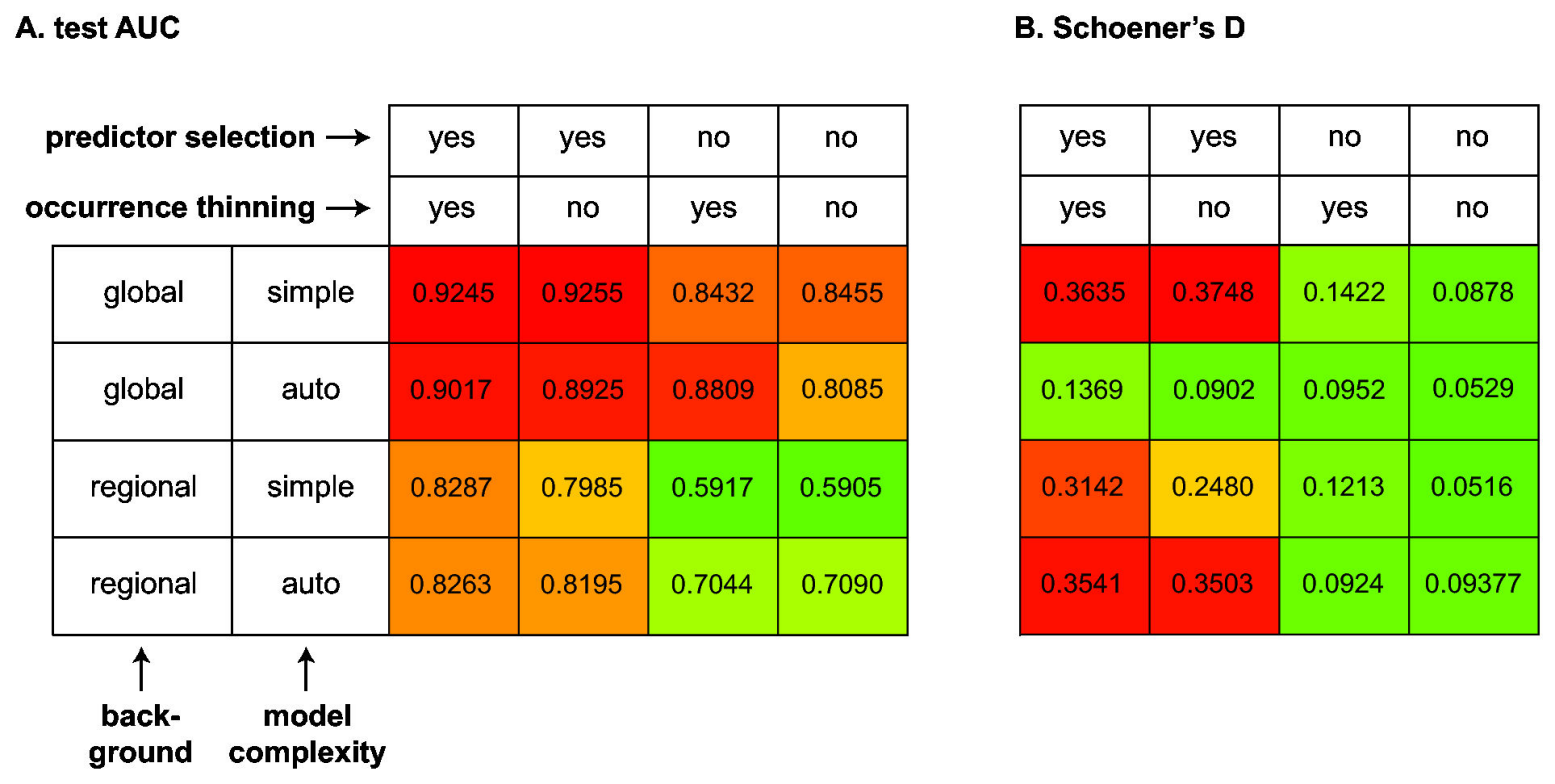
Impact of modeling choices on the transferability of SDMs. The transferability of models is approximated by test AUC (panel A) and the global niche overlap (Schoener’s D, panel B). Columns and rows represent the combinations of the four factors that were varied in our experimental design and are identical in both panels. The values are also plotted as colors along a color gradient to permit rapid visual assessment of the important factors, with warmer colors indicating higher values. Each AUC value in panel A represent the average of the AUC_native-invaded_ and AUC_invaded-native_ for the corresponding condition.

Our multifactorial experiment showed that reducing the number of predictors, based on our surveying method, yielded much better models with higher test AUCs (Figure 3A) and Schoener’s D (Figure 3B) than models with the full set of eight predictors. This is clearly visible in both figures: the leftmost two columns of both panels of the figure have warmer colors than the rightmost two columns. A Wilcoxon signed-rank test (WSRT) indicated that the difference in test AUC and Schoener’s D between matching models is significant (p = 0.0078 in both cases, N = 8).

With test AUC as the measure of transferability (Figure 3A), the two upper rows had warmer colors than the lower two rows, suggesting better performance of models that use global background samples compared to models in which background samples are restricted to the region in which the model is trained. This pattern was not present in the Schoener’s D values (Figure 3B), where models with global background and auto-features had remarkably low values of D, and the WSRT outcomes conflicted strongly (p = 0.0078 for AUC, p = 0.9453 for Schoener’s D, N = 8). The higher AUC with global backgrounds may thus be a consequence of the sensitivity of AUC to background choice rather than an actual increase in predictive power with global backgrounds.

Model complexity and occurrence thinning did not have a large effect on transferability between regions. However, the second row in Figure 3B shows substantially lower Schoener’s D for a set of models with auto-features compared to the same set of models with enforced simple models (the row above). This difference was not present for the regional background case (3rd vs. 4th row).

### Overall predictive performance of SDMs

Models built with occurrences from throughout the native and invaded ranges have considerably higher predictive power than models trained on one range and projected onto the other (WSRT, p = 0.0156 and 0.0078 for AUC_global_ vs. AUC_native→invaded_ and AUC_global_ vs. AUC_invaded→native_ respectively, N = 8, for pairs with global background only). These models’ test AUC values, calculated on 50% random test occurrences from throughout the range, are all close to 1 (Table 1), indicating strong overall predictive power. The predictive performance of models based on pooled occurrences from native and invaded regions barely differ between conditions, indicating that models built with occurrences from both ranges are less sensitive to choices made during the modeling process (Table 1).

**Table 1:** Predictive performance of models built with occurrences from native and invaded ranges as a function of choices made in the modeling process.

**occurrence thinning**	**predictor selection**	**model complexity**	**performance (test AUC)**
yes	no	simple	0.975
yes	no	auto	0.990
yes	yes	simple	0.982
yes	yes	auto	0.988
no	no	simple	0.972
no	no	auto	0.991
no	yes	simple	0.974
no	yes	auto	0.992

The overall predictive performance, as measured by the test AUC, is very high and the factors have only a minor influence on the outcome. All models compared in this table use the same set of 10,000 background points (global, equal area).

#### An SDM for *Caulerpa* ** *Cylindracea*

**Figure 4:**
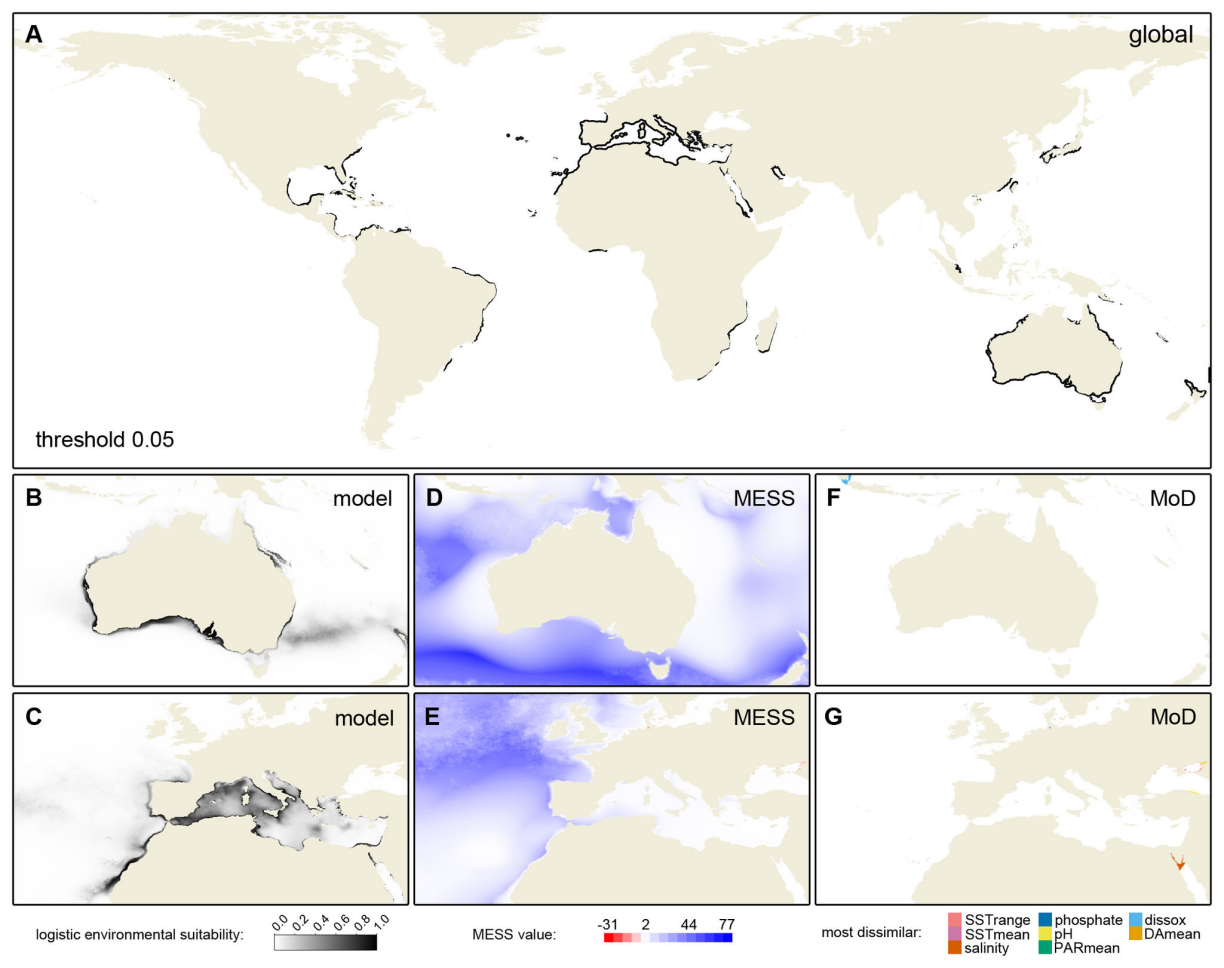
Species distribution model for *Caulerpacylindracea* . Panel A shows global areas predicted to have suitable macroecological conditions for the species. This map uses a threshold for Maxent’s logistic suitability corresponding to the 10% training presences (threshold = 0.053) and predictions are plotted only for coastal areas (less than 7 pixels from shore), with predictions in the open ocean masked. Panels B and C show the continuous logistic model output for the native and invaded ranges, respectively. The corresponding multivariate environmental similarity surface (MESS) maps are shown in panels D and E, and the most dissimilar (MoD) variables in those areas that require extrapolation are shown in panels F and G.

The various SDMs with high predictive power were visually similar, and we present environmental suitability maps of one of the top-scoring models in Figure 4. The global map, which uses a threshold to indicate predicted suitable areas, clearly highlights large parts of the coasts of Australia (native region) and the Mediterranean Sea (invaded region) as having suitable macroecological conditions. In addition, the model predicts suitable environmental conditions along the East Coast of the USA, parts of the Caribbean region, the tropical to warm-temperate coast of Brazil, parts of the coasts of Madagascar and Southeast Africa, as well as Taiwan and the main Japanese islands.

Within the native region (Australia, Figure 4B), the model predicts suitable macroecological conditions along almost the entire coast of southern Australia, including northern Tasmania, the west and east Australian coasts except for a region in SE Queensland, and parts of the north coast, where some regions had intermediate predicted suitability. These predictions are a considerable extension of the presently known range of the species (Figure 1C), and high environmental suitability is predicted in the various embayments of southern Australia where the species has recently established and become a conspicuous member of the benthic community. The multivariate environmental similarity surface (MESS) map is positive in almost the entire range (Figure 4D, blue colors), which indicates that the conditions present in the region were observed in the training data and gives extra credibility to the model prediction. Given that the MESS map is mostly positive the "most dissimilar" (MoD) variable map is nearly blank (Figure 4F).

In the invaded region (Figure 4C), the model also predicted beyond the known occurrences of the species (Figure 1A), including Portugal, the NW of Spain and the NW of Africa. In the East, suitable macroecological conditions were inferred for the northern Red Sea, although the MESS map indicates that there is extrapolation beyond observed environmental conditions (Figure 4E), with the MoD map highlighting the (high) salinity occurring in the northern Red Sea as the most dissimilar variable.

The entire Maxent run including input data and all outputs is available for examination on FigShare (http://dx.doi.org/10.6084/m9.figshare.681723). Besides showing the main results presented here in more detail, this resource also allows examining limiting factors and exploring the components of the prediction for particular sites with Maxent’s explain tool.

## Discussion

Our results have implications for the invasion biology of *Caulerpacylindracea* as well as the more general question of how best to model the distribution of species introduced outside their native range. We will first highlight the effects of the different distribution modeling practices on model transferability and performance, as well as some limitations of the procedures described here. Then we will discuss the meaning of our SDMs for the spread of *C* *. cylindracea* in Europe and Australia.

### Building more reliable SDMs of introduced species

Niche conservatism is a central assumption when extrapolating correlative SDMs of introduced species to an area outside the bounds of training occurrences. The poor predictive power of SDMs trained in the native range and projected onto the invaded range that has been observed in many studies led to the conclusions that ecological niches can shift in association with introductions outside of the native range (e.g. [19,21,71-73], but see 6). In interpreting such niche shifts, it is important to realize that correlative models estimate a species’ realized niche and that, as a consequence, observed niche shifts do not necessarily reflect physiological changes (i.e., modifications of the fundamental niche). In other words, the perceived niche shift can result from two different realizations of the same fundamental niche in different areas, and it has been argued that this scenario is more parsimonious than that in which the fundamental niche changes [9,74]. However, changes in the fundamental niche of introduced species are certainly possible [4,15].

Regardless of whether niche shifts observed in correlative SDMs are a consequence of changes in the realized or fundamental niche, it would certainly be useful to have a set of procedures that improve the predictive power of SDMs outside the training range in order to inform conservation planning and decision making. The methods used here were applied hoping they would improve the transferability of the SDMs of introduced species built using the popular presence-only method Maxent. We found that reducing the number of predictor variables drastically improved the transferability of our SDMs. Limiting the model complexity, reducing geographic sampling bias by occurrence thinning and choosing a global background had comparably small effects.

The effect of the choice of predictors has long been known to have a drastic effect on the transferability of SDMs of introduced species (e.g., [4,9,19,20,21]). The method used here, which surveys all combinations of variables for the native as well as the invaded region, attempts to identify variables that are likely to be of global rather than regional significance. Models based on the set of variables identified by this approach were more transferable than models with a more comprehensive set of variables, irrespective of whether reciprocal test AUC or Schoener’s D were used to measure transferability. Although the use of procedures to select predictors and model complexity in an automated manner is common practice in many types of modeling including niche modeling [75-77], to our knowledge such approaches have not been used commonly in combination with Maxent. However, we do acknowledge that such predictor selection methods are no substitute for physiological knowledge of the organism [78], and here they were used to further refine a set of predictors that was already reduced from the full Bio-ORACLE dataset based on what we know are important factors determining algal growth.

Previous studies have also shown that reducing the complexity of models to fit smoother responses yields the best correspondence to physiological knowledge and as such, the models achieve better overall performance and have higher transferability [7,10,11,79]. For these reasons, the use of simple environmental response surfaces to avoid overfitting has been recommended for SDMs of invasive species [4,7,11]. Generally, the complexity of maximum entropy models is adjusted by using L_1_ regularization [68], which varies along a continuous scale and has been used in other studies aimed at improving the performance of Maxent SDMs [10]. We chose to use a simple dichotomy between Maxent’s auto-features versus the use of only linear and quadratic features to keep the experimental setup simple. Our results did not show a meaningful difference between the transferability of models built under both conditions and thus we did not observe the improvement of predictions with simpler models that other studies have [7,10]. This can probably be attributed to the fact that model complexity does not differ much between the two conditions in our experimental setup: the auto-features condition only differed in having hinge features in addition to the linear and quadratic features used in the "simple" condition. Nonetheless, we follow previous authors in their conclusion that correlative models with smooth responses will generally outperform those with complex responses. This is especially true if the number of occurrence points used to build models is large, because this increases the potential for overfitting. Since the identification of suitable predictors and an appropriate level of model complexity are related to one another, it may be advisable to integrate these two into a single procedure as commonly done in classical model selection procedures [75].

The use of thinned occurrences generally resulted in SDMs with better transferability, but the effect was not significant in a Wilcoxon signed-rank test and small compared to that obtained from predictor selection. Nonetheless, we anticipate that this approach may be useful in situations where the geographical bias is stronger than in our dataset and/or in situations with stronger spatial autocorrelation in the environmental grids. Other approaches that have been proposed to deal with geographic bias in occurrence records are to introduce the same sort of bias in the background points by specifying a target-group background, using bias grids in Maxent, or through application of trend surface analysis [7,25,26]. Various statistical approaches to address spatial autocorrelation have also been used [13]. In our case study, the background selection had a rather limited effect on the transferability of SDMs and in this context, it is worth noting that there were differences between the transferability results depending on whether they were measured as test AUC or as Schoener’s D. The difference was most pronounced for models with global backgrounds and auto-features (compare second row in Figure 3A with second row in Figure 3B). It is well known that AUC is sensitive to background choice, with larger backgrounds inflating AUC values while not yielding more informative models [26,80,81]. Our observation of higher AUC values for global backgrounds compared to regional backgrounds, which was not paralleled in Schoener’s D, is completely in line with this. As such, for comparisons of the transferability of models built with different backgrounds, we suggest the use of Schoener’s D rather than test AUC. Regarding the transferability of models as a function of the background selection, a previous study concluded that using background in reachable areas provides a "less risky prediction space" [7]. Our experiments did not confirm this conclusion but suggested that transferability (as measured by Schoener’s D) is indifferent to the choice of background.

From the results discussed above it is clear that the usefulness (i.e., the predictive power) of reciprocal niche models is quite variable and strongly depends on the choices made. While they barely outperform random models under some conditions (some test AUC < 0.6 in Figure 3A), making the right choices outlined above improves the predictive power of models trained in one range and projected onto the other (0.90 < test AUC < 0.93 for the best models, Figure 3A). Nevertheless, if distribution data are available from both the native and invaded ranges, it is advisable to build models from a combined set of occurrences. For our data, models based on combined occurrences outperformed reciprocal models (test AUC > 0.99 for best models). In this case, it is appropriate to use test AUC to compare performance, as all these models are built and evaluated using identical background points. Similar conclusions regarding the better predictive power of models using combined native-invaded datasets were reached in studies of other species (e.g., [4,82]). Our results also suggest that the combined data have the advantage of being more insensitive to the modeling choices that need to be made, but this generalization should be verified with other case studies.

### Potential limitations

Besides discussing the performance of the various methods applied, it is also useful to point out their assumptions and potential caveats.

Firstly, our case study had the advantage of having relatively large sets of occurrence records for the native as well as the invaded range. In many cases, however, one will want to build reliable predictive models for species that were recently introduced and for which only a few occurrences have been recorded in the invaded range. How could a suitable set of predictors be identified in this case? Our approach relied on having sufficient data to identify those variables with predictive power in both geographic regions separately and combined. As an alternative, one could first identify the predictors achieving predictive power in the native range and subsequently compare the frequency distribution of those variables between samples from the native and invaded ranges with the aim of avoiding variables for which the invaded samples are outside of the range of values of native samples. It may also be beneficial to upweight the scarce samples from the invaded range in the model-building step. It is worth noting that we used an essentially arbitrary threshold to retain predictor variables, i.e. they had to be present in 60% or more of the top-scoring models for at least two out of three regions (Figure 2). This approach was chosen because variables important in multiple regions are more likely to be of global importance, and secondly because the 60% threshold resulted in a halving of the number of predictors. However, this raises the question of how these criteria influence the results and whether more objective criteria could be used. The evaluation of all these ideas as well as other possible approaches is an attractive avenue for further research.

Our general approach towards increasing the transferability of SDM does not make explicit assumptions about whether or not a niche shift between ranges is present, or if it is, whether it is situated at the level of the fundamental or the realized niche. The ideal scenario is that there are no niche shifts between the populations and transferability is not an issue. However, if a niche shift is present, our predictor reduction approach will eliminate those predictors that have poor predictive power in one or both ranges, regardless of whether any changes in predictive power between regions are due to differences in the realized or fundamental niche. While we expect that eliminating predictors that have regional rather than general relevance will be sound in a majority of cases, there are scenarios imaginable where this will not work. For example, if the correlation structure of predictor variables differs between regions, an indirect variable (i.e. one that does not affect the distribution but is correlated with another one that does affect it) may be identified as important in both regions but have very different response curves in both areas and thus lead to poor transferability. Similarly, variables that are directly relevant to the distribution may differ systematically between regions, decreasing the transferability of the SDMs built from them [74].

Even though it can be expected that the distance-based thinning will improve most models, this may not always be the case. In fact, this procedure may discard useful data when regions of dense sampling coincide with steep ecological gradients over short geographic distances. Also, if sampling reflects population densities, geographic autocorrelation of records can add a potentially desirable quantitative aspect to the model. This will, of course depend on the specific goal and the dataset being studied.

Finally, our evaluation of methods is based on a single case study, and there are no guarantees that our results will extrapolate to other introduced species. A logical next step is to apply these methods to a range of suitable case studies. The time since the introduction and dispersal potential of the species should be prime criteria in selecting species to further test these methods. Species that were introduced a long time ago and have had the chance to disperse widely in the invaded range are more likely to have spread through their entire potential niche and thus make good case studies.

An additional approach towards testing the degree to which these methods can be generalized, as well as to explore the various other questions raised in the discussion, is to carry out simulation experiments. Simulation is a powerful tool for testing the logical consistency of ideas as well as the efficiency and reliability of methods. They have not been widely used to evaluate presence-only SDM methods, although there appears to be a trend towards their increased use in recent years [17,74,79,83-87]. Besides identifying the circumstances in which niche modeling algorithms perform well and those in which they are more likely to fail, simulation is a powerful tool to assess the effectiveness of procedures such as those described here. Such insights would obviously be beneficial to the whole SDM field.

### Invasion and spread of *Caulerpa* *Cylindracea*

The distribution model presented for *C* *. cylindracea* predicted potential expansions in the invaded range along East Atlantic coastlines of Europe and Africa as well as a substantial potential expansion along the southern coast of Australia (Figure 4A). Admittedly, the logistic values in Maxent lack a clear-cut interpretation [88] and determining thresholds for presence-only SDMs is not an exact science [89,90]. Based on several thresholds tested (e.g. 10-percentile training presence, equal training sensitivity and specificity), the inferred range boundaries are quite far beyond the known occurrences of the species (Figure 1 vs. Figure 4A). This suggests that our current knowledge may underestimate the potential range of this species in these areas. In the Mediterranean and East Atlantic region, the species has only been present for only about 20 years and, despite the species’ relatively rapid colonization rate [91], it is likely that it has not reached its distributional limits yet. In Australia, the native area of the species, it was known best from the Western Australian coast [48]. However, the recent observations of invasive populations of this species along the southern coast, where it did not previously occur (reference [46] and pers. obs.), prompted us to generate SDMs for this species in order to investigate whether the species could potentially colonize more of the coast. Our models do indeed suggest that the macroecological conditions are highly favorable and that *C* *. cylindracea* could colonize the entire southern coastline of Australia. Besides these potential expansions in regions where the species is present already, several other coastlines are predicted to be suitable environment where the species could establish if it were to be introduced (Figure 4A).

Needless to say our models only incorporate macroecological predictor variables. Besides this, the microhabitat, as well as possible biotic interactions, also need to be favorable for the species to establish itself in the areas that are predicted to be suitable. In its native range, *C* *. cylindracea* is usually found on rocky substrata close to the low-tide mark but in more tropical locations (NW Australia and the Great Barrier Reef) it is typically found growing on sand in lagoons and around reefs. In the Mediterranean Sea, it has been found between 1 and 60 meters depth, on all types of hard and soft substrata and in different communities, with the only exception being unstable sandy substrata [29]. A number of studies have studied the microhabitat preferences of the species in some detail in the Mediterranean, showing that it thrives on rocky substrata among other macroalgae as well as in dead seagrass beds [92-94], and that it tolerates near-bottom orbital velocities below 15 cm s^-1^ [93]. In summary, the species occurs in a wide range of common microhabitats, so it is likely that it could establish in the great majority of areas predicted by our SDM if there are no biotic interactions inhibiting its settlement and expansion.

The correlative model from this study can also be used to inform experimental studies on the physiological tolerances of *C* *. cylindracea* . Even though we have not shown or discussed detailed response curves in the main paper, these are available as supplementary materials on FigShare (http://dx.doi.org/10.6084/m9.figshare.681723). Most correspond to our expectations based on physiological knowledge of other algae, including other *Caulerpa* species [95], but some do not. For example, the correlative model indicates that the species is mainly found in phosphate-poor waters with the response curve rapidly dropping at concentrations over 0.4 µmol L^-1^. Studies on other species indicate that macroalgae have an increasing response curve for macronutrients and that low rather than high concentrations may be limiting seaweed species in nature [96-99]. This suggests that our correlative model may be misled in this case. It is also interesting to note that models built from occurrences in the native range predicted a much broader range of suitable temperatures than models from occurrences in the invaded range. More specifically, the model from invasive occurrences has a response curve that peaks at ca. 20^°^C, dropping off quickly at higher temperatures. The curve from a model with native occurrences also peaks at ca. 20^°^C, but drops much more gently at higher temperatures. Whether this should simply be interpreted as an indication that warmer areas are yet to be colonized in the invaded range (i.e. that the model is biased towards colder temperature due to the current distribution), or that the introduced strain has a reduced range of temperature tolerance compared to the native population, remains to be investigated. To further characterize the most relevant features determining the species’ range, it would be informative to evaluate the gradients of predictors occurring across the inferred range boundaries, and put those to the test in physiological experiments.

## Conclusions

In order for Maxent presence-only SDMs to be useful in predicting and managing introduced and invasive species, a number of problems related to their accuracy and transferability have to be overcome. The methods introduced, explored and evaluated here aim to improve the situation. Reducing the set of predictors to those anticipated to be of global significance resulted in a strong improvement of SDM transferability, with occurrence thinning, model complexity and background choice having relatively minor effects. If available, occurrences from the native and invaded regions should be combined, as this yields the best-performing models and apparently reduces their sensitivity to choices made in the modeling process. We also presented an SDM of *Caulerpacylindracea* that achieves very high predictive power, illustrating the applicability of these methods in the marine realm for which comparably little niche modeling has been done [100]. The procedures introduced here are available for further evaluation with other case and simulation studies, which should provide further insights into the degree to which our results can be generalized. We hope and anticipate that they will form a useful strategy to improve predictive SDMs and in turn, help to better inform environmental decision makers.

## Supporting Information

Figure S1 Model surveying results indicating qualitatively similar results when analyses are carried out with global or regional backgrounds.
(PDF)
