# Supplementary material for: Improving Transferability of Introduced Species’ Distribution Models: New Tools to Forecast the Spread of a Highly Invasive Seaweed
Source: PLoS One. 2013 Jun 28;8(6):e68337. doi: 10.1371/journal.pone.0068337 (PMC3732097; doi:10.1371/journal.pone.0068337)
Supplement: Figure S1 — Model surveying results indicating qualitatively similar results when analyses are carried out with global or regional backgrounds. (PDF) [file pone.0068337.s001.pdf]

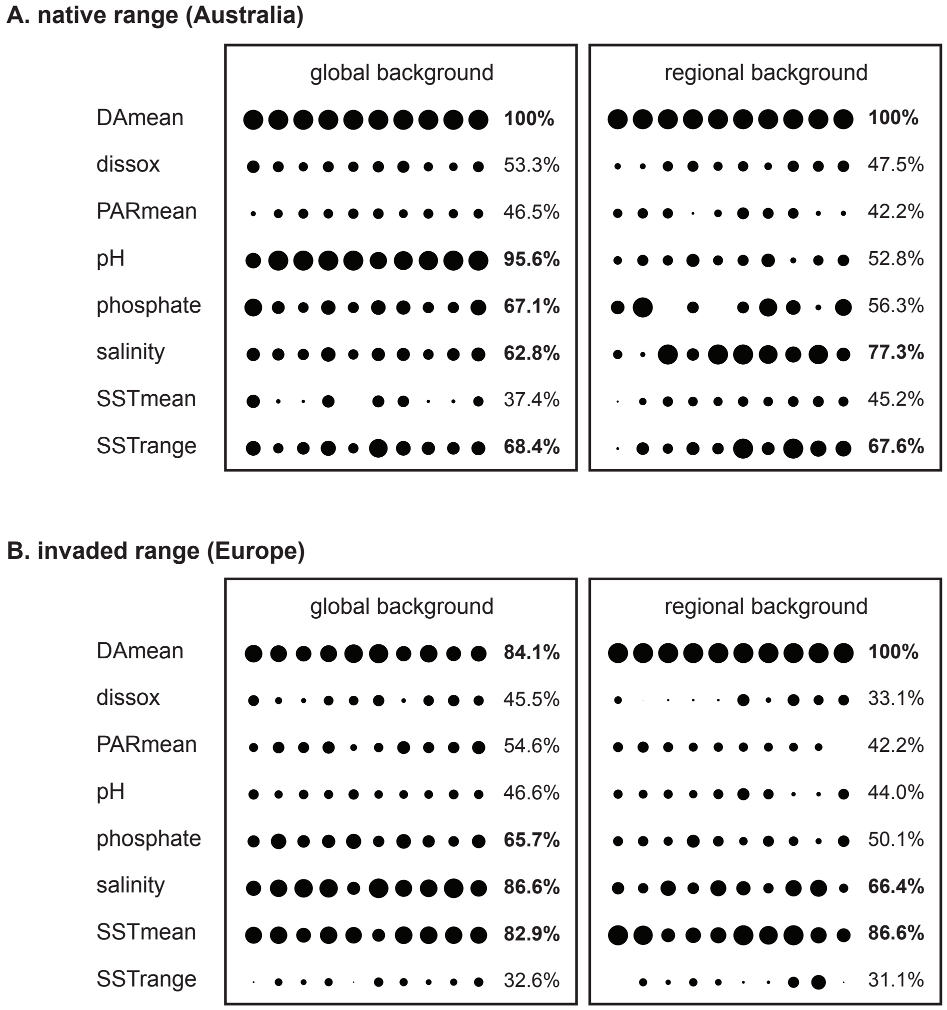

Fig. S1: Model surveying results indicating qualitatively similar results when analyses are carried out with global or regoinal backgrounds.
